# Activation of β-Catenin by Oncogenic PIK3CA and EGFR Promotes Resistance to Glucose Deprivation by Inducing a Strong Antioxidant Response

**DOI:** 10.1371/journal.pone.0037526

**Published:** 2012-05-25

**Authors:** Luca Cardone, Alberto Bardelli, Vittorio Enrico Avvedimento

**Affiliations:** 1 Department of Biology and Cellular and Molecular Pathology, School of Medicine, University Federico II of Naples, Naples, Italy; 2 Laboratory of Molecular Genetics, Institute for Cancer Research and Treatment, University of Turin Medical School, Turin, Italy; 3 FIRC Institute of Molecular Oncology (IFOM), Milan, Italy; Catholic University Medical School, Italy

## Abstract

Glucose is an essential fuel for cell survival and its availability limits aberrant cellular proliferation. We have hypothesized that specific cancer mutations regulate metabolic response(s) to glucose deprivation (GD). By means of somatic knock-in cellular models, we have analyzed the response to glucose deprivation in cells carrying the frequent *^delE746-A750^EGFR*, *^G13D^KRAS* or *^E545K^PIK3CA* cancer alleles. We demonstrate that, in mammary epithelial cells, glucose has an essential antioxidant function and that these cells are very sensitive to GD. Conversely, isogenic cells carrying the *^delE746-A750^EGFR* or the *^E545K^PIK3CA*, but not the *^G13D^KRAS* allele, display high tolerance to GD by stimulating the expression of anti-oxidant genes (*MnSOD* and *catalase*). This adaptive transcriptional response is mediated by the activation of WNT/β-catenin and FOXO4 signalling. Our data highlights a new functional synergism between oncogenic EGFR and PIK3CA with WNT/β-catenin conferring high tolerance to oxidative stress generated by nutrient deprivation.

## Introduction

Glucose is a fundamental cellular fuel for the generation of ATP and NADH through glycolysis and the mitochondrial oxidative phosphorylation; it is also an essential component of the pentose phosphate shunt pathway (PPP) that leads to the production of NADPH used to synthesize reduced glutathione, a potent intracellular antioxidant. Thus, glucose deficit is likely to produce energetic dysfunction and oxidative stress.

Glucose limitation is a common stress during tumor progression: glucose concentration acts as an energetic barrier against the aberrant cellular proliferation of pre-malignant cells [1]; moreover, an energetic deficit occurs in certain areas of solid tumors where glucose and oxygen deficiencies are generated by the unstable tumor microenvironment or ischemia [1],[2]. Thus, cellular adaptation to sub-optimal nutrients concentrations appears as a relevant phenotype that cancer cells acquire during tumor progression. A key open issue is to identify the specific genetic alteration(s) that drive the metabolic adaptation of cancer cells to glucose deficiency. Recently, the *^G13D^KRAS* and *^V600E^B-Raf* oncogenes have been associated with a resistance to low glucose and *^G13D^KRAS* mutations occur in cells upon selection in low glucose environment [3]. In addition, tumors carrying deletions of the tumor suppressor PTEN, a lipid phosphatase, are resistant to caloric restriction *in vivo* [4].

Epidermal Growth Factor Receptor (EGFR) and the phosphatidylinositol 3-kinase (PI3K), are oncogenes frequently mutated in human cancers. PI3Ks are a family of lipid kinases that phosphorylate the 3-OH group on phosphatidylinositols in the plasma membrane. This leads to the recruitment to the cell membrane and activation of the protein Ser/Thr-kinase AKT. The PI3K/AKT signaling cascade is critical in cancer development since it controls the activity of fundamental cell fate regulators and promotes cell survival and growth. Activating mutations in *PIK3CA*, the gene encoding the p110α catalytic subunit or inactivating mutations of *PTEN*, have been identified in a variety of solid tumors [5], [6], including colorectal, breast and endometrial cancers. Notably, three recurrent oncogenic “hotspot” mutations include the majority of somatic *PIK3CA* mutations. Two of these mutations, the E542K and the E545K, occur in the helical domain, and the third mutation, H1047R, affects the kinase domain [7]. All three mutations result in enhanced lipid kinase activity.

EGFR is a receptor tyrosine kinase involved in the control of DNA synthesis, cell proliferation, migration and adhesion [8]. Upon binding with extracellular ligands and dimerization, EGFR leads to the activation of multiple intracellular signaling pathways, such as the PI3K/AKT, the MEK/ERK and the JAK/STAT pathways. EGFR overexpression by gene amplification or by EGFR activation have been associated with several cancers, including lung and breast cancer and *glioblastoma multiforme* [9], [10]. A specific point mutation L858R- and short in-frame deletions in exon 19 account for approximately 90% of the mutated cases [11]. The in frame deletion E746-A750 in exon 19 induces a ligand independent EGFR dimerization and activation [12].

To date, we have limited information about the interference of the specific cancer mutations of EGFR and PIK3CA with metabolic responses of cells exposed to glucose deprivation. To gather information on this issue, we have investigated the cellular response to glucose deprivation (GD) in cells carrying the *^delE746-A750^EGFR*, the *^E545K^PIK3CA* or the *^G13D^KRAS* mutations. For these studies, we have implemented a panel of isogenic cell lines generated by targeted homologous recombination to introduce (knock-in) a cancer allele in the genome of human somatic cells [13]. The derivative cells express the cancer alleles under the control of their endogenous promoter, thus allowing the study of the mutated proteins under physiological conditions relative to the expression levels and transcriptional regulation.

Our studies reveal that, in mammary epithelial cells, GD induces a drop in the ATP content, a significant reduction of the cellular antioxidant power resulting in oxidative stress and ultimately, cell death. In contrast, isogenic cells carrying *^delE746-A750^EGFR* or *^E545K^PIK3CA* alleles, upon GD, engage antioxidant strategies, by increasing the expression of *MnSOD* and *catalase* genes that attenuate the oxidative stress. The activation of such adaptive transcriptional response is mediated by WNT signals through the action of β-catenin and FOXO4 transcription factors.

## Results

### The *^delE746-A750^EGFR* and the *^E545K^PIK3CA* cancer alleles confer resistance to GD

We implemented a panel of isogenic cells generated by targeted homologous recombination (Knock-in) of *^delE746-A750^EGFR*, *^E545K^PIK3CA* or *^G13D^KRAS* cancer alleles in immortalized human mammary epithelial cells (HME) [13]. The expression of cancer alleles affects the regulation of downstream signaling pathways as confirmed by serum deprivation experiments (Figure S1): in fact, serum starvation elicited a dose-dependent reduction of phosphorylation of AKT(Ser473), of EGFR(Tyr1068), and ERK1/2(Thr202/Tyr204) in wild type HME cells, while isogenic clones, expressing the oncogenes, did not specifically reduce the phosphorylation of the same substrates (Figure S1).

**Figure 1:**
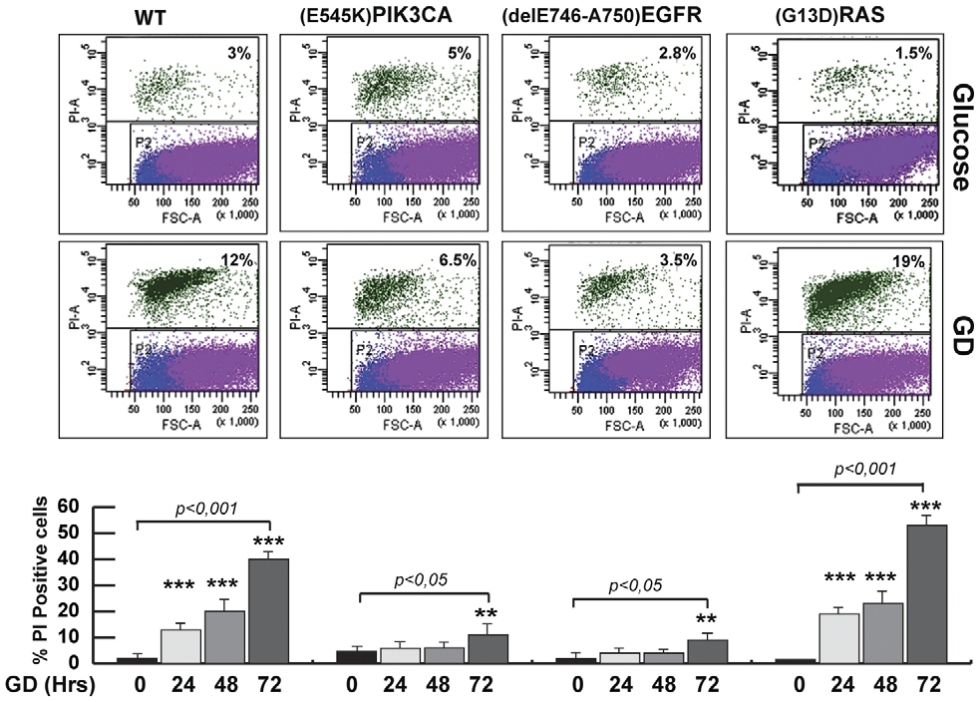
HME cells carrying the *^delE746-A750^EGFR* or the *^E545K^PIK3CA* allele are resistant to GD-induced cell death. Wild type or isogenic clones carrying *^delE746-A750^EGFR* or *^E545K^PIK3CA* or *^G13D^KRAS* alleles were glucose-starved (GD) for the indicated hours and the percentage of dead cells were quantified by FACS analysis of propidium iodide positive cells. Graphs report the average of three independent experiments ± SD (*t-*test, **p<0.05 or ***p<0.001, Not treated Vs GD-treated cells). The diagrams are representative of a FACS analysis performed 24 hours after GD.

We then investigated cell survival in response to GD of wild type and the isogenic lines expressing the mutant alleles. Prolonged GD affects cell cycle and cell viability [14], [15]. FACS analysis revealed that GD induced cell death in wild type cells, while isogenic clones expressing the ^E545K^PIK3CA or the ^delE746-A750^EGFR mutated proteins were resistant to GD. In contrast, the presence of *^G13D^KRAS* allele specifically led to higher sensitivity to GD (Figure 1). Similar results were observed in independently generated isogenic HME clones (Figure S2). To rule out that the observed differences in cell viability between wild type cells and the isogenic derivatives were an artefact due to the procedures used to generate the cellular model, we analyzed isogenic HME cells generated through the homologous recombination of the wild type alleles of *EGFR* or *PIK3CA* genes, here referred to as PIK3CA_cnt and EGFR_cnt. GD induced cell death of these control cells as well as in wild type HME cells (Figure S2). These data indicate that resistance to GD-induced death is specifically conferred by the activating *^delE746-A750^EGFR* or *^E545K^PIK3CA* mutations.

### The oncogenic variants control ATP levels and GSH/GSSG ratio in response to GD

**Figure 2:**
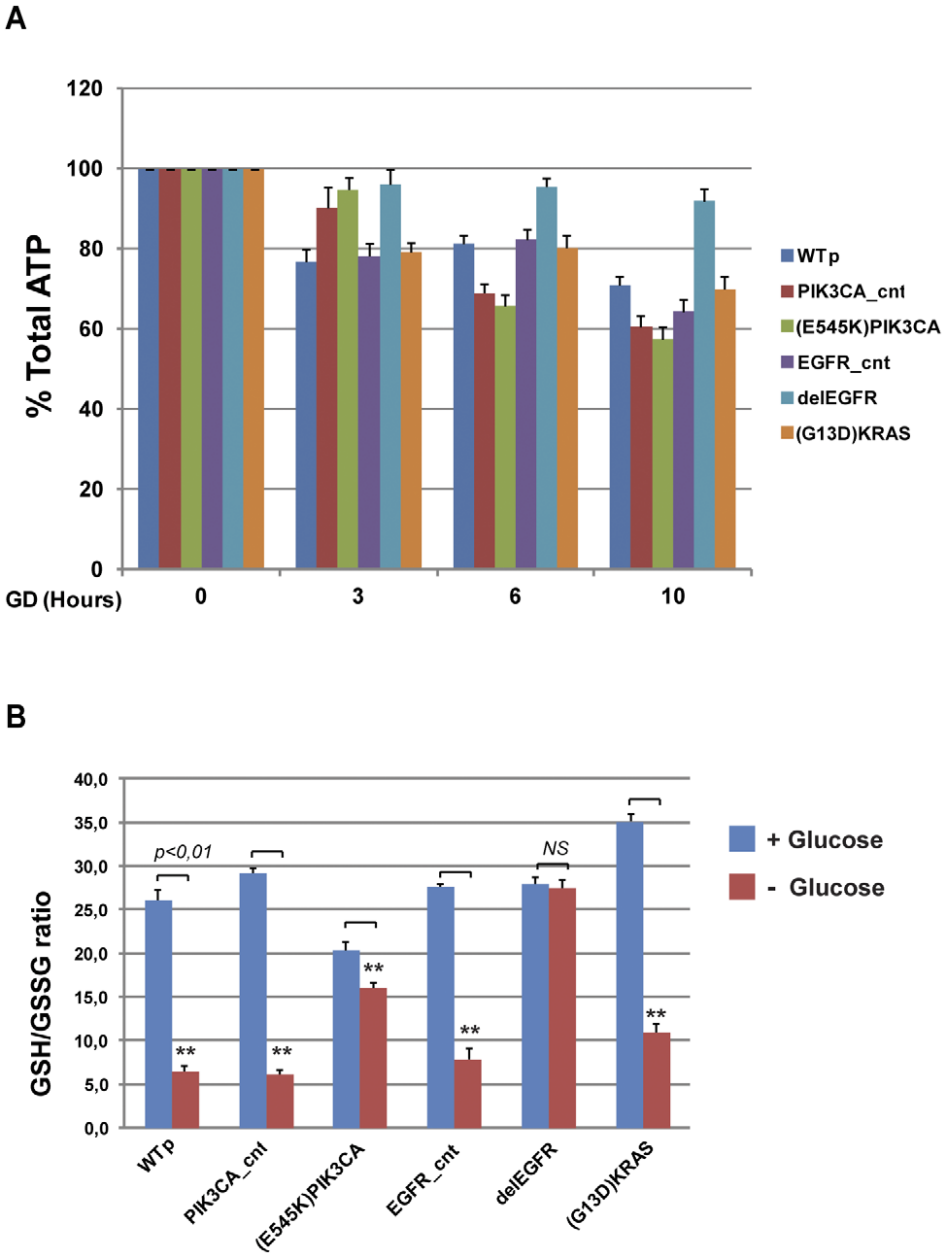
ATP content and GSH/GSSG ratio in wild type or oncogene-expressing HME cells after glucose deprivation. (A) Total ATP content after GD in wild type HME cells and isogenic clones carrying *^delE746-A750^EGFR*, *^E545K^PIK3CA* or *^G13D^KRAS* cancer alleles. At the indicated time, cells were harvested and the total ATP content was analyzed; ATP amount was normalized for the number of nuclei. Results (mean ± SD, n = 3) are expressed as percentages of the ATP amount respect to T = 0. WTp: wild type HME parental cells; PI3K_cnt and EGFRdel_cnt are HME control cells carrying the wild type alleles obtained through somatic homologous recombination (see text for details). (B) Reduced and oxidized glutathione ratio (GSH/GSSG) in wild type, control and oncogenes-carrying cells after GD. Cells were glucose starved for 10 hours, and then the GSH/GSSG ratio was measured. Results report the average of eight independent experiments ± SD (*t-*test, **p<0.01, Not treated Vs GD-treated cells. *ns*: not significant).

Glucose is a fundamental cellular energy supply for ATP production. Moreover, glucose also fuels the pentose phosphate pathway (PPP), the metabolic process that stimulates the anabolism and generates cellular NADPH and supports the production of reduced glutathione (GSH), the most important cellular antioxidant. Thus, GD is expected to induce both energetic and oxidative stress. To evaluate the metabolic effects elicited by GD in HME wild type and isogenic clones, we measured total ATP content and the ratio between reduced and oxidized glutathione (GSH/GSSG ratio) following GD. Time course analysis revealed that wild type HME and isogenic clones carrying *^G13D^KRAS* or *^E545K^PIK3CA* oncogenes displayed a significant time-dependent reduction of the ATP levels following GD; in contrast, isogenic clones carrying the *^delE746-A750^EGFR* retained 100% of ATP content up to 10 hours after treatment (Figure 2A). Moreover, GD induced a significant reduction of the GSH/GSSG ratio in wild type HME cells, in *^G13D^KRAS*-carrying cells and in isogenic control cells but NOT in clones carrying *^delE746-A750^EGFR* and *^E545K^PIK3CA* alleles (Figure 2B). These data show that glucose is essential in mammary epithelial cells to maintain redox homeostasis and that some oncogenic mutations specifically compensate redox unbalance induced by glucose deprivation

### GD induces cell death through oxidative stress

**Figure 3:**
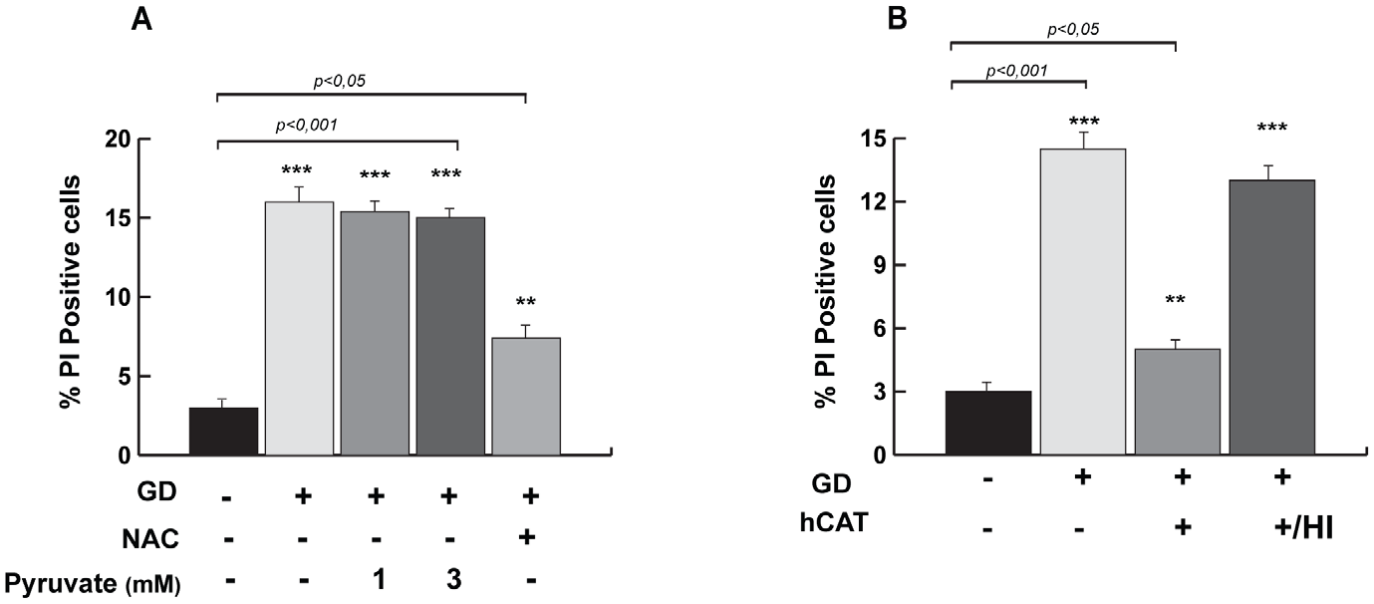
NAC and Catalase confer resistance to GD-induced cell death. (A) Antioxidant but not pyruvate treatment inhibits GD-induced cell death. Wild type HME cells were glucose starved for 36 hours with or without 5 mM N-acetyl-L-Cysteine or sodium pyruvate at the indicated concentrations. The percentage of dead cells was quantified by FACS as in Fig 1. The data are derived on the average from at least three independent experiments ± SD (*t-*test, **p<0.05 or **p<0.001). (B) Purified human catalase treatment induces cellular resistance to GD-induced cell death. Wild type HME cells were glucose starved for 36 hours with or without 12 hours of pre-treatment with purified human catalase (600 U/ml) or heat inactivated catalase (HI). The percentage of dead cells was quantified as in (A).

The results in Figure 2 indicated that, in wild type cells, GD elicited a drop of the intracellular GSH/GSSG ratio that reduced the intracellular antioxidant power and induced a redox stress. To analyze the relevance of the oxidative stress generated by GD, we measured the effects of antioxidants on GD-induced cell death. Pre-treatment of wild type HME cells with N-Acetyl-L-Cysteine, which increases the GSH pool and enhances ROS scavenging, prevents cell death induced by GD (Figure 3A). Notably, under the same conditions, pre-treatment of the cells with sodium pyruvate, did not affect GD-induced cell death, indicating that the energetic stress, resulting from ATP drop, was indeed not the major driver of GD-induced cell death (Figure 3A). As NAC contributes also to H_2_0_2_-scavenging by increasing the GSH intracellular pool, we assessed the relevance of H_2_0_2_ concentration in GD-induced cell death. Our data show that treatment with purified human catalase, an H_2_0_2_-scavenger enzyme, inhibited GD-induced cell death in wild type HME cells (Figure 3B). Taken together, these data indicate that glucose has an essential antioxidant role in mammary epithelial cells.

### AMPKα is a sensor of GD-induced oxidative stress

**Figure 4:**
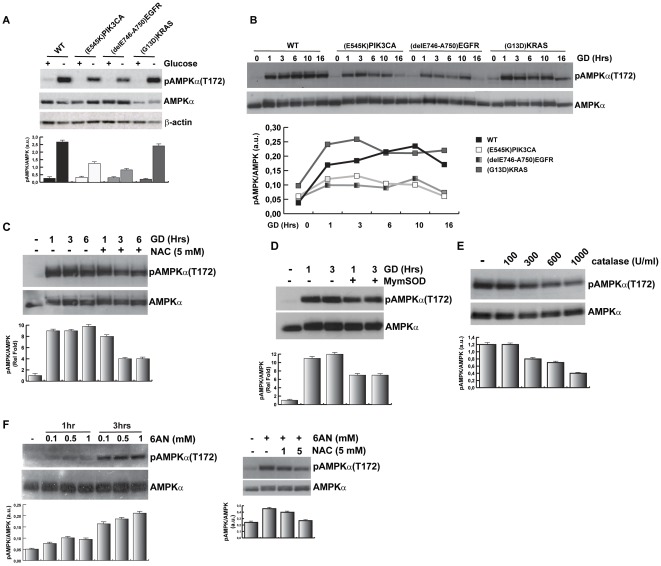
GD-induced oxidative stress control AMPKα activation. (A) Wild type HME or isogenic cells carrying the indicated cancer alleles were glucose deprived for 10 hours and equal amounts of protein lysates were assayed by immunoblot with the indicated antibodies. The graph reports the densitometry analysis of the pAMPKα/AMPKα_total_. (B) Time course analysis of AMPKα phosphorylation after GD. Wild type HME and isogenic cells carrying *^delE746-A750^EGFR* or *^E545K^PIK3CA* or *^G13D^KRAS* alleles were glucose starved for the indicated time. Total proteins were analyzed by immunoblot as indicated. The graph reports the densitometry analysis of the pAMPKα/AMPKα_total_. *Wild type*: black square; *^E545K^PIK3CA*: white square; *^delE746-A750^EGFR*: gray square; *^G13D^KRAS*: dark gray square. Data are representative of three independent experiments that gave similar results. (C and D) Treatment with antioxidants attenuates GD-induced pAMPKα(T172). Wild type HME cells were pre-treated with N-acetyl-L-Cysteine (C) or mimetic SOD (D) 1 hour before glucose starvation. Proteins extracts were analyzed by immunoblot with anti-pAMPKα(T172) and anti-AMPKα antibodies. The graph reports the densitometry analysis of the pAMPKα/AMPKα_total_; average from at least three independent experiments ± SD are reported as relative fold to not-treated cells. (E) Catalase treatment reduces GD-induced pAMPKα. Wild type HME cells were treated with catalase at the indicated concentrations and harvested after 3 hours of GD. Equal amount of protein lysates were analyzed by immunoblot. (F) G6PDH inhibitor induces AMPKα(T172) phosphorylation through ROS generation. Wild type HME cells were treated as indicated with 6-AN in glucose- and serum-containing media (left panels) or were pre-treated with NAC before 6-AN treatment (right panels) and total protein extracts were analyzed by immunoblot with anti-pAMPKα(T172) and anti-AMPKα antibodies. Histograms in E and F report the densitometry analysis of the pAMPKα/AMPKα_total._ Average from at least three independent experiments ± SD were plotted.

The AMP-activated protein kinase (AMPK) is a key molecular sensor and regulator of the cellular response to glucose deprivation [16], [17]. To further understand to what extent glucose deprivation elicits a metabolic stress in wild type HME and in isogenic clones carrying oncogenes, we studied the activation of AMPKα by measuring the phosphorylation of AMPKα(T172), a specific marker of kinase activation [16], [17]. Our data show that AMPKα(T172) was highly phosphorylated upon 10 hours of GD in wild type cells and *^G13D^KRAS* expressing cells, whereas this phosphorylation was attenuated in isogenic HME cells carrying the *^E545K^PIK3CA* or the *^delE746-A750^EGFR* cancer alleles (Fig. 4A). Similar results were observed in independently generated isogenic HME clones (Figure S3A). Moreover, the observed attenuation was specifically induced by the oncogenes, since control cells displayed a robust activation of AMPKα(T172) phosphorylation after GD treatment (Figure S3B). The attenuation of AMPKα phosphorylation by cancer alleles was further confirmed by time course analysis of AMPKα(T172) phosphorylation after GD (Figure 4B).

**Figure 5:**
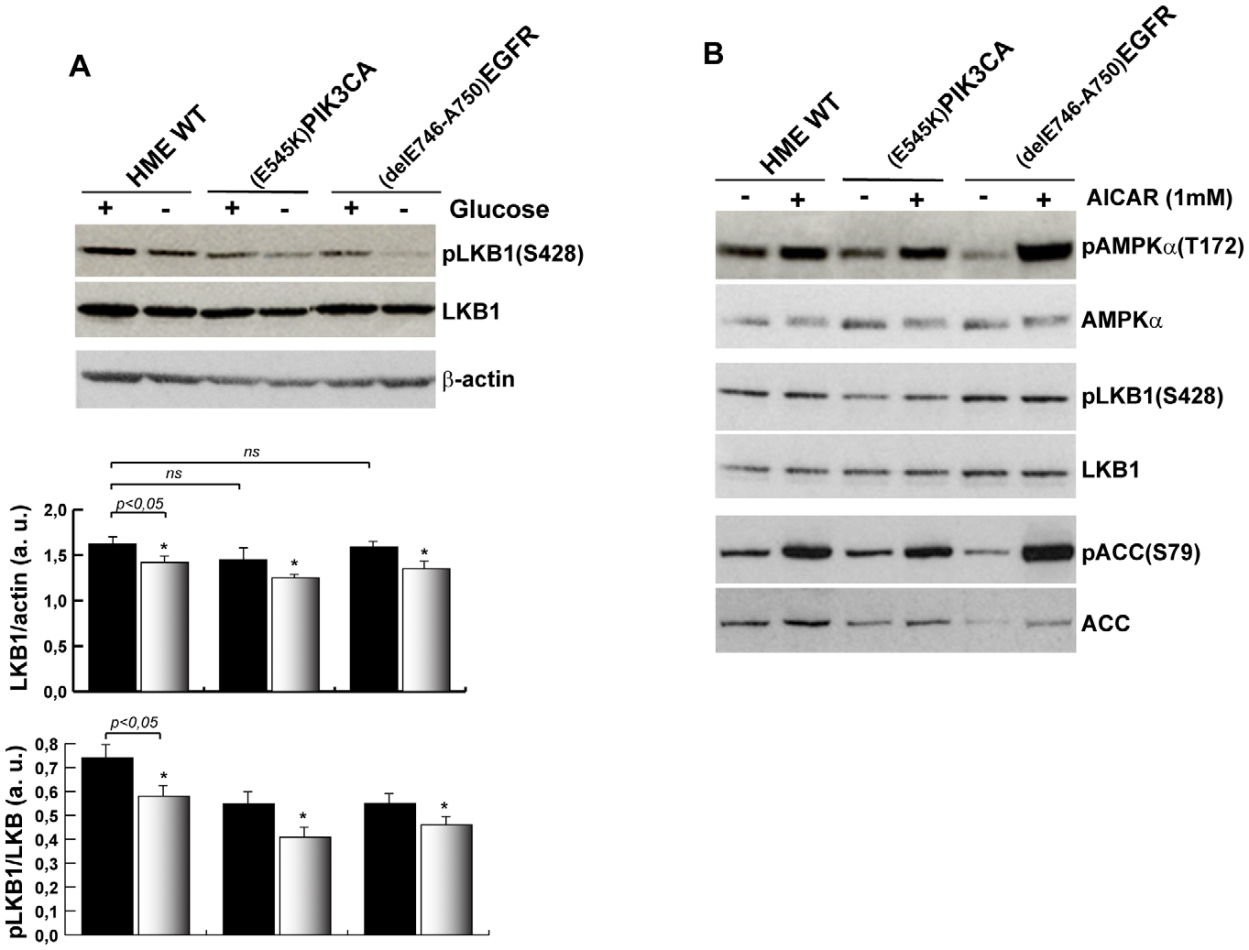
Cells expressing *^delE746-A750^EGFR* or *^E545K^PIK3CA* cancer alleles have functional AMP/LKB1/AMPK sensor machinery. (A) Regulation of LKB1 kinase in HME cells after GD. Equal amounts of total protein lysates of wild type or isogenic cells were analyzed by immunoblot with antibodies against LKB1 and pLKB1(Ser428); β-actin was used as loading control. The graph indicates the densitometry analysis of the data derived from at least three independent experiments (*t-*test, *p<0.05, Not treated Vs GD-treated cells. *ns*: not significant). (B) AICAR treatment increases pAMPKα(T172) in wild type and in oncogene-carrying cells and does not influence LKB1 expression. The indicated isogenic cell lines were treated for 2 hours with 1 mM AICAR and total protein lysates were analyzed by immunoblot with the indicated antibodies.

AMPK is a general stress sensor that can be activated by AMP or by multiple intracellular signals such as calcium [18], free fatty acids [19] and reactive oxygen species (ROS) [20]. To link the oxidative stress induced by GD to AMPK activation, we pre-treated wild type HME cells before GD with the antioxidant NAC and we analyzed the AMPKα(T172) phosphorylation. Immunoblot analysis of protein extracts shows that NAC attenuated GD-induced phosphorylation of AMPK (Figure 4C). Similar results were obtained by pre-treating the cells with a SOD mimetic compound that is able to neutralize cellular superoxide (Figure 4D). Finally, pre-treatment of wild type HME cells with purified human catalase reduced also GD-induced AMPK phosphorylation (Figure 4E). The antioxidant activity of glucose is dependent on its ability to stimulate G6PDH activity and to support the PPP: in fact, the inhibition of G6PDH by the specific inhibitor 6-aminonicotinamide (6AN) was sufficient to induce oxidative stress and to phosphorylate AMPK in the presence of glucose. 6AN-induced AMPKα phosphorylation was controlled by ROS since it was inhibited by the pre-treatment of cells with NAC (Figure 4F). These data show that GD induces a significant oxidative stress that contributes to AMPK phosphorylation. In cells carrying activated oncogenes, this circuitry is not efficient. It is possible that the oxidative stress is attenuated in these cells, as suggested by Figure 2. However, it is also likely that AMPK is inhibited by oncogenic ERK-dependent signals through increased phosphorylation of the major AMPK activating kinase, LKB1, which, when phosphorylated at Serine 428, inhibits AMPK [21], [22]. To discriminate between these two possibilities we measured the phosphorylation of LKB1(S428) after GD in wild type HME and isogenic clones. Western blot analysis showed that LKB1 levels were comparable in wild type HME and in isogenic clones (Figure 5A). GD induced a reduction of total LKB1 protein both in wild type and isogenic cells carrying *^E545K^PIK3CA* or *^delE746-A750^EGFR* alleles. By using specific antibodies against phospho-Ser428LKB1, we found that GD reduced phospho-LKB1(S428) levels in wild type HME cells as well as in isogenic clones (Figure 5A). Moreover, the treatment with AICAR, an AMP analogue, efficiently induced AMPKα(T172) phosphorylation in wild type HME cells as well as in isogenic clones carrying the *^delE746-A750^EGFR* and *^E545K^PIK3CA* alleles (Figure 5B). Importantly, AICAR treatment, in contrast to GD, did not downregulate LKB1 and phospho-LKB1(S428) levels (Figure 5B), supporting the hypothesis that other mechanisms, in addition to the AMP/ATP ratio, control the LKB1/AMPK complex after exposure to GD. Collectively, these data demonstrate that mammary epithelial cells expressing the *^delE746-A750^EGFR* and the *^E545K^PIK3CA* oncogenes have a functional AMP/LKB1/AMPKα sensor circuitry and that the attenuation of AMPK activation depends on the control of oxidative homeostasis.

### *^delE746-A750^EGFR* and *^E545K^PIK3CA* oncogenes specifically induce antioxidant enzymes in response to GD

**Figure 6:**
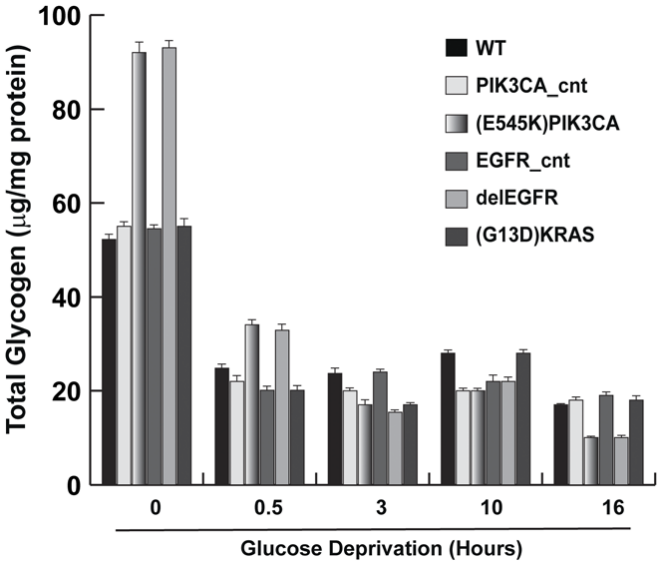
Total glycogen content in oncogene-expressing HME cells after glucose deprivation. Total glycogen content after GD in wild type HME cells and isogenic clones carrying *^delE746-A750^EGFR, ^E545K^PIK3CA* or *^G13D^KRAS* cancer alleles. At the indicated time, cells were harvested and total glycogen content was analyzed. Results (average of three independent experiments ± SD) are expressed as glycogen content normalized for protein mass. WT: wild type HME cells; PIK3CA_cnt and EGFR_cnt are HME control cells carrying the wild type alleles (see text for details).

A simple mechanism explaining the resistance of oncogene-carrying cells to GD is the production of endogenous glucose, for example from glycogen storage. Determination of total glycogen levels indicated that HME cells carrying the *^delE746-A750^EGFR* and *^E545K^PIK3CA* alleles contain higher total glycogen compared to isogenic wild type or control HME (Figure 6). Notably, 30′ after GD, we observed a rapid and strong decrease of glycogen content in both control and oncogene-carrying cells; after 30 minutes of GD, glycogen content declined in all cell lines, although steeper in oncogene-carrying cells than controls. Also, the pharmacological inhibition of glycogenolysis with a glycogen phosphorylase inhibitor (CP91149) did not significantly affect the viability of oncogene-carrying cells after GD (data not shown). These data indicate that glycogen storage was not responsible for the resistance to GD of oncogene-carrying cells.

**Figure 7:**
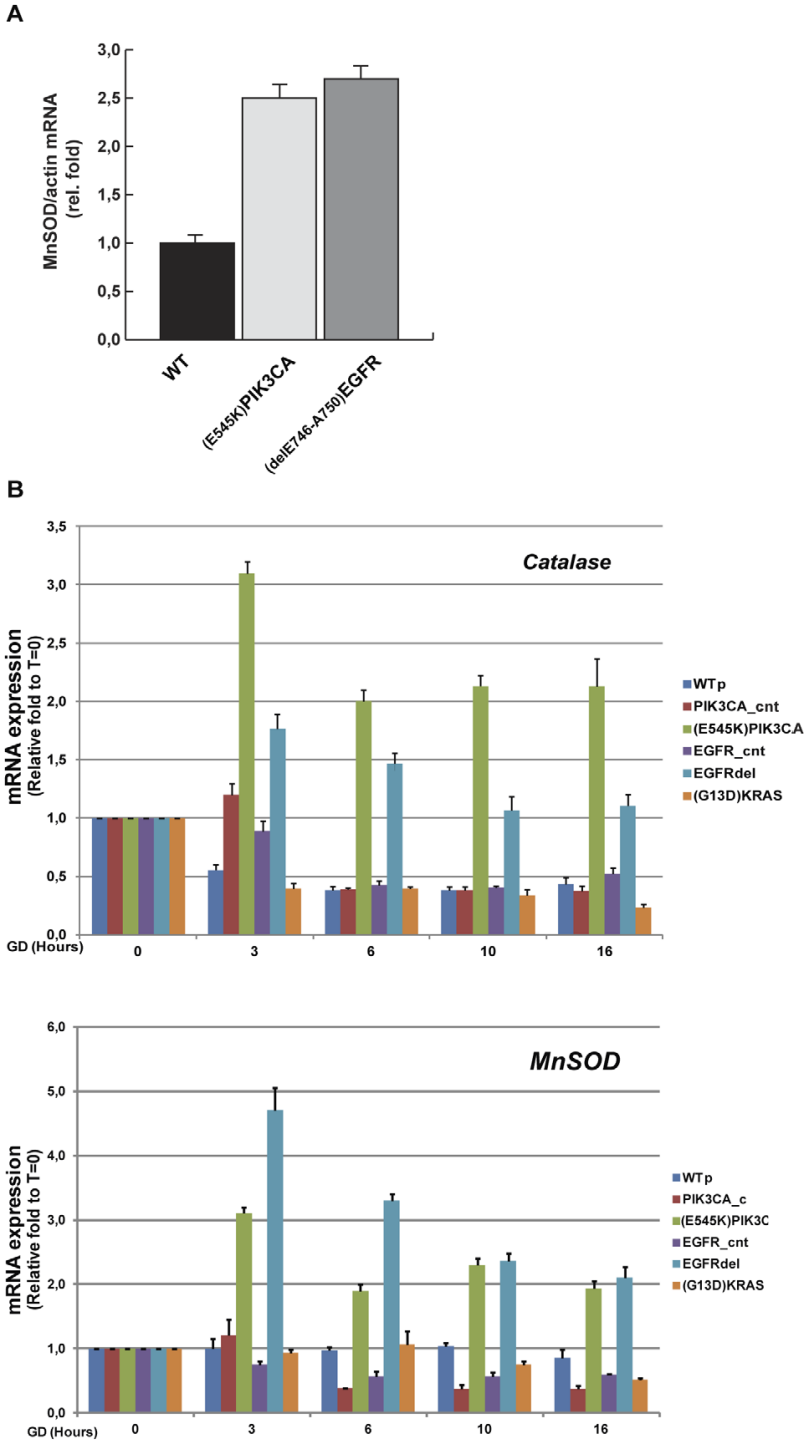
GD induces the transcription of antioxidant enzymes in cells carrying the *^delE746-A750^EGFR* or the *^E545K^PIK3CA* alleles. (A) Upregulation of *MnSOD* expression by oncogenes. Total RNA was harvested from wild type HME or the indicated isogenic clones. The expression of *MnSOD* normalized to β-actin mRNA was analyzed by quantitative real time PCR. Data (means ± SD) indicate fold relative to wild type cells. (B) Wild type or the indicated isogenic HME cells were glucose starved and RNA was harvested at the indicated time points. The relative expression of the *MnSOD* or *Catalase* genes, normalized to β-actin mRNA, was analyzed by quantitative real time PCR. Data show means ± SD.

We next tested the possibility that resistance to GD may depend on antioxidant strategies. An efficient antioxidant response relies on the rapid changes of expression of antioxidant enzymes. To this end we analyzed the expression of relevant antioxidant genes. Quantitative real time-PCR analysis revealed that HME cells expressing the *^delE746-A750^EGFR* or *^E545K^PIK3CA* alleles showed a two fold increase of *Manganese Superoxide dismutase* (*MnSOD*) mRNA compared to wild type cells (Figure 7A). Moreover, HME clones expressing selectively EGFR and PIK3CA oncogenes displayed a robust expression of *Manganese Superoxide dismutase* (*MnSOD*) and *catalase* mRNAs following GD (Figure 7B). On the other hand, *^G13D^KRAS*-expressing cells did not stimulate the expression of these antioxidant genes (Figure 7B). Western blot analysis on total protein extracts confirmed that oncogene-carrying clones have significant higher levels of MnSOD protein compared to wild type cells (Figure S4).

### β-catenin and FOXO4 are involved in the oncogenes-driven responses to GD

**Figure 8:**
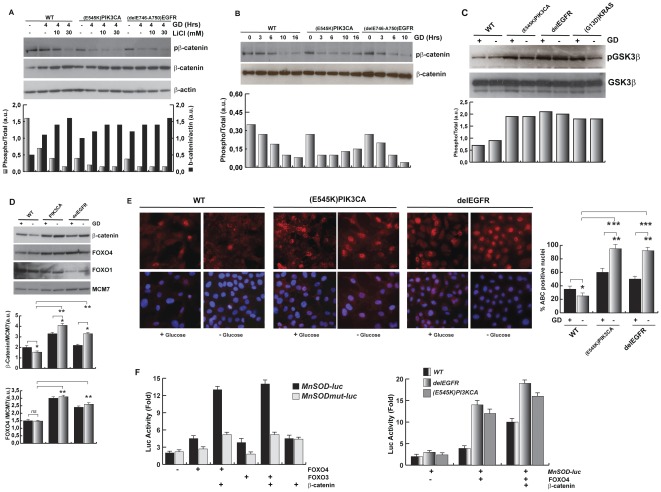
Upregulation of FOXO4 and β-catenin by EGFR and PIK3CA oncogenes contributes to *MnSOD* expression in response to GD. (A) GD controls β-catenin phosphorylation and stability. Wild type HME or oncogene carrying cells were glucose deprived for 4 hours in the presence or not of lithium chloride and equal amounts of proteins were assayed by immunoblot. The anti-pβ-catenin antibody recognizes the phosphorylated Ser33,37 and Thr41 residues of β-catenin. The graph reports the densitometry analysis of the pβ-catenin/β-catenin (gray bars) and the β-catenin/β-actin (black bars). (B) Time course analysis of β-catenin phosphorylation after GD. HME clones were GD for the indicated time. Total proteins were analyzed by immunoblot as indicated. The graph reports the densitometry analysis of pβ-catenin/β-catenin. (C) Regulation of GSK3β phosphorylation in response to GD. The indicated HME clones were glucose starved for 6 hours and total protein extracts were analyzed by immunoblot as indicated. pGSK3β antibody recognizes specifically the phospho-Ser9 residue of GSK3β. The graph reports the densitometry analysis of the pGSK3β/β-catenin signals. (D) Regulation of nuclear β-catenin and FOXO4 accumulation by oncogenes in response to GD. Wild type HME or isogenic cells carrying *^delE746-A750^EGFR* or *^E545K^PIK3CA* cancer alleles were glucose starved for 5 hours and equal amounts of nuclear protein extracts were assayed by immunoblot as indicated. MCM7 antibody recognizes the nuclear minichromosome maintenance protein 7 and is here used as loading control. Graphs report the densitometry analysis of indicated protein/MCM7 signals ratio. Results report the average of three independent experiments ± SD (*t-*test, **p<0.01; *p<0.05). (E) Intracellular localization of active β-catenin (ABC) after GD exposure. Wild type HME or isogenic cells carrying the *^delE746-A750^EGFR* or the *^E545K^PIK3CA* cancer alleles were glucose-starved for 5 hours. Cell were fixed and stained with a specific anti active, not phosphorylated β-catenin antibody (Red) and DAPI (Blue) for the nuclear staining and analyzed by fluorescence microscopy. Graphs report the percentage of β-catenin positive nuclei (average ± SD of 10 different fields containing at least 40 cells/field. *t-*test, ***p<0.001, **p<0.01, *p<0.05). The exposure time was kept constant through the images analysis. (F) β-catenin contributes to *MnSOD* promoter activation under GD. (Left graph) Wild type HME cells were transfected with vectors expressing the indicated proteins together with a wild type MnSOD promoter luciferase-reporter (−3340+1*MnSOD-luc)*, or with a mutant derivative which contains mutated FOXO binding sites (−3340+1mut*MnSOD-luc*) and analyzed after 8 hours of GD. Data represent means ± SD derived from four independent experiments. (Right graph) Wild type HME cells or isogenic clones expressing the delE746-A750EGFR or the E545KPIK3CA allele were transfected with vectors expressing the indicated proteins in presence of wild type MnSOD promoter luciferase-reporter and analyzed after 8 hours of GD. Data represent means ± SD. The efficiency of transfection was normalized by the cotransfection of CMV-Renilla luciferase reporter.

To find the specific transcriptional network induced by the oncogenes following GD, we have searched for transcription factors that stimulate *Catalase* and *MnSOD* gene expression. These genes are known targets of the Forkhead transcription factors (FOXOs) [23], [24] and β-catenin, a key effector of the WNT pathway [25], [26]. Although the PI3K/AKT-dependent signaling has been recognized as a negative regulator of FOXOs, FOXO4 and β-catenin proteins can accumulate into nucleus and act as transcriptional sensors of oxidative stress independently of the presence of growth factors [26], [27]. Based on these observations, we studied the regulation of β -catenin and FOXO4 after GD in wild type HME cells and in isogenic clones carrying oncogenes. Since stability and nuclear localization of β-catenin are negatively controlled by a GSK3β kinase-dependent phosphorylation of Ser33,37 and Thr41 residues of β-catenin, we assayed the phosphorylation of these residues in response to GD. Western blot with specific antibodies indicated that phosphorylation of the Ser33,37 and Thr41 of β-catenin was rapidly inhibited in response to GD in oncogenes-carrying cells but not in wild type cells (Figure 8A and 8B). Ser33,37 and Thr41 phosphorylation of β-catenin in wild type cells under GD was mainly dependent on GSK3β kinase activity since exposure to the GSK3β inhibitor, lithium chloride, eliminated the phosphorylation of these residues of β-catenin (Figure 8A). These data suggest that oncogenic EGFR and PI3K selectively inhibit GSK3β during GD. Accordingly, immunoblot analysis showed that the levels of phosphorylated GSK3β(S9), an inhibitory site phosphorylated by AKT, was higher in oncogene-expressing cells exposed to GD (Figure 8C).

Since active β-catenin is targeted to the nucleus, we monitored β-catenin nuclear accumulation in response to GD. Our data show that HME cells carrying-oncogenes accumulate significant nuclear β-catenin in response to GD compared to isogenic wild type cells (Figure 8D). To further document the activation of β-catenin, we performed immmunoflorescence analysis by using specific antibodies recognizing active, not-phosphorylated, β-catenin. Our data indicate that nuclear localization of active β-catenin is increased in HME cells expressing the *^delE746-A750^EGFR* or the *^E545K^PIK3CA* alleles compared to the isogenic wild type cells; after exposure to GD, active β-catenin robustly accumulate into nucleus of oncogenes-carring cells, more efficiently than wild type cells. These data demonstrate that the ^delE746-A750^EGFR and the ^E545K^PIK3CA proteins stimulate β-catenin activation and targeting to the nucleus following exposure to glucose deprivation (Figure 8E). We next analyzed the expression of FOXO4: western blot analysis demonstrated that oncogenes-carrying cells displayed a 2-fold increase of nuclear FOXO4 compared to wild type cells, independently of GD (Figure 8D); this effect was specific to FOXO4, since nuclear FOXO1 was indeed reduced in cells carrying activated EGFR and PI3K pathways (Figure 8D). However, we observed a reduction of nuclear FOXO1 in response to GD in wild type cells but not in isogenic clones carrying oncogenes, suggesting that oncogenic EGFR and PIK3CA also stabilized FOXO1 in response to GD (Figure 8D).

To further investigate whether FOXO4 and β-catenin cooperate to activate the expression of antioxidant genes in response to GD in HME cells, we used a *MnSOD* promoter reporter assay previously described [25], [26]. β-catenin was able to enhance FOXO4-dependent and independent transcription of the *MnSOD* promoter in wild type HME cells under GD (Figure 8F, left graph), indicating that *MnSOD* promoter can be also activated by stimuli FOXO4-independent, but β-catenin-dependent (Figure 8F, left graph). As expected, *MnSOD* promoter reporter assay was significantly stimulated in HME cells carrying the *^delE746-A750^EGFR* or the *^E545K^PIK3CA* oncogenes compared to wild type cells (Figure 8F, right graph).

### The GSK3β/FOXO4/MnSOD axis enhances the survival of mammary epithelial cells exposed to GD

**Figure 9:**
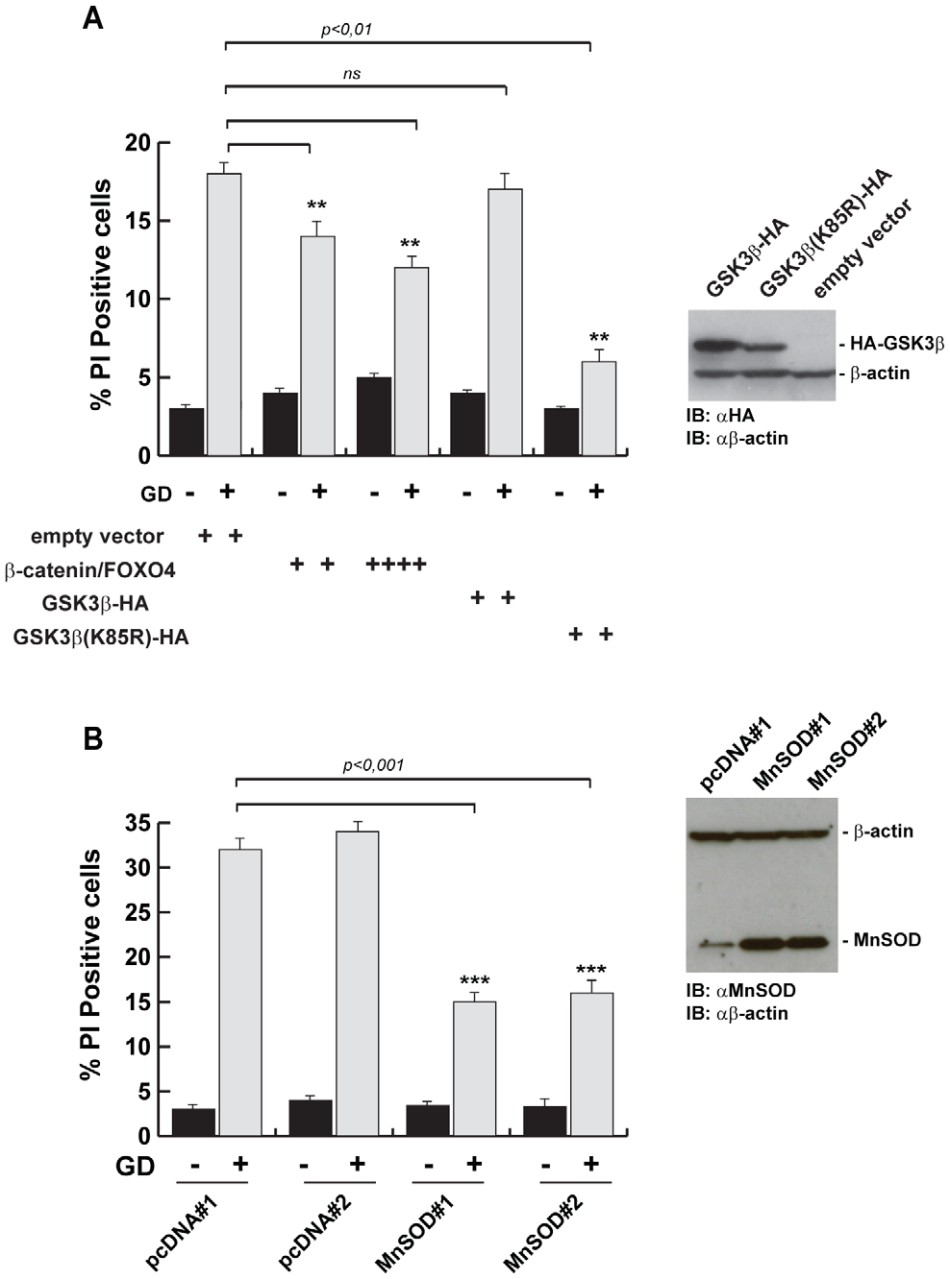
The GSK3β/β-catenin/MnSOD axis promotes resistance to GD-induced cell death. (A) Wild type HME cells were cotransfected with equal amounts of the indicated expression vectors. 24 hours after transfection, cells were glucose-starved for 48 hours and the percentage of dead cells was quantified by FACS analysis of propidium iodide positive cells. Graphs report the average derived from three independent experiments ± SD (*t-*test, **p<0.01). The inset shows an anti-HA immunoblot of total protein extracts from cells transfected with the HA-GSK3β or HA-GSK3β(K85R) vectors and used for the GD experiments. β-actin was used as loading control. (B) Wild type HME cells stably transfected with an empty vector (pcDNA#1 and pcDNA#2) or with an expression vector for MnSOD (MnSOD#1 and MnSOD#2) were exposed to GD for 60 hours and the percentage of dead cells were quantified by FACS analysis of propidium iodide positive cells. At least three independent empty vector- or MnSOD-transfected clones were analyzed and gave similar results. The inset represents an immunoblot analysis showing the expression of MnSOD in total protein extracts from two representative clones used for the experiments. β-actin was used as loading control. Graphs report the average derived from three independent experiments ± SD (*t-*test, **p<0.01 or ***p<0.001, empty vector Vs MnSOD).

To functionally link the GSK3β and β-catenin axis with the response to glucose deprivation, we co-expressed β-catenin and FOXO4 in wild type HME cells and monitored cell death after 48 hours of exposure to GD. Our data demonstrate that the expression of β-catenin and FOXO4 improves resistance to GD of wild type HME cells (Figure 9A). Moreover, we also demonstrate that the expression of the GSK3β(K85A) mutant - a GSK3β kinase dominant negative - protects wild type cells from GD-induced death (Figure 9A). Expressing wild type GSK3β kinase did not induce the protective effect. These data underline the importance of the constitutive, oncogenic activation of EGFR/PI3K/GSK3β signaling in protecting and selecting cells during GD.

To directly link the MnSOD expression with resistance to GD, we generated wild type HME cell lines stably expressing the antioxidant MnSOD enzyme. Upregulation of MnSOD expression was sufficient to protect the cells from GD-induced cell death (Figure 9B). These results indicate that the increased MnSOD expression is an important component of oncogene-induced resistance to GD in cells carrying the *^delE746-A750^EGFR* or *^E545K^PIK3CA* cancer alleles.

All together, these data show that the oncogenic inactivation of GSK3β by the ^delE746-A750^EGFR or ^E545K^PIK3CA cancer proteins significantly increases nuclear β-catenin pool in response to GD and enhances β-catenin- and FOXO4-dependent expression of genes involved in antioxidant stress response.

## Discussion

### An essential antioxidant role of glucose in mammary epithelial cells

In mammary epithelial cells the metabolic checkpoint for glucose concentration is based on ROS homeostasis. GD induces a significant reduction of the GSH/GSSG ratio, a severe oxidative stress and, ultimately, cell death. GSH level is controlled by PPP and depends on activation of NADPH oxidase and SOD. Activation of G6PDH and glutathione peroxidase in combination with NADPH oxidase and SOD maintains stable GSH levels [28]. In the absence of glucose and under low ATP levels, this process is inefficient and the GSH/GSSG ratio decreases, leading to rise of ROS levels. ROS generated by GD induce the phosphorylation and activation of AMPKα. Similarly, 2-Deoxy-D-glucose, a not-hydrolysable glucose analogue, stimulates AMPKα(T172) phosphorylation by a ROS-mediated mechanism (LC, unpublished data and [29]). Thus, ROS generated by GD act as metabolic intermediates able to activate AMPK. HME cells expressing the *^delE746-A750^EGFR* and *^E545K^PIK3CA* mutants are resistant to GD-generated oxidative stress and show reduced AMPK activation.

We have found that the resistance strategies involve, at least in part, the activation of antioxidant enzymes such as MnSOD and catalase. The upregulation of MnSOD is adaptive since it increases the ROS neutralization as well as the rate of GSH production [28]. High basal levels of MnSOD expression has been reported associated to invasive and highly aggressive breast cancer [30], [31] and this may indicate the high ability of tumor cell to adapt to nutrient deprivation. Since the EGFR and PIK3CA are among the most frequently activated oncogenes in cancer, we suggest that the specific anti-oxidant transcriptional program driven by these oncogenes protects and favours the selection of the cells carrying the mutated proteins. Our data offer a mechanistic explanation for the selection and the reproductive success of such cells under environmental metabolic stress. We suggest that MnSOD and catalase expression may represent a common anti-oxidant mechanism in pre-cancerous cells harbouring activating mutation of the EGFR- and PI3K-dependent pathways. The ability to tune metabolic enzymes and pathways to survive under nutrient stress may represent a relevant and common phenotype of cancer cells. In this framework, we believe that also the hypertrophy of the serine synthesis pathway in breast cancer cells [32], [33] reflects the ability of cancer cells to continuously adapt to different metabolic needs.

### Oncogenes and glucose metabolism: not just a matter of addiction in tumor progression

Specific metabolic changes occur during tumor development and allow cellular adaptation to the unstable tumor microenvironment. One of the most prominent metabolic changes in cancer cells is the high glycolytic rate in the presence of oxygen, a phenomenon known as the Warburg effect [1], [34], [35]. As consequences of the Warburg effect, cancer cells show increased glucose needs and higher sensitivity to glucose deprivation compared to normal cells, a phenomenon known as glucose addiction [35], [36]. In cancer cells, the expression of constitutive active AKT [37], [38], KRAS [3], [39], [40] or the activation of the mTOR pathway [14], [41] has been associated with an increased Warburg effect and higher sensitivity to glucose deprivation. Here, we demonstrate that in not-transformed mammary epithelial cells GD elicits cell death and that the expression of oncogenic EGFR and PIK3CA confers resistance rather than sensitivity to glucose deprivation. Moreover, we did not observe a significant increase of the glycolytic index in oncogenes-carrying clones compared to wild type HME (data not shown). This apparent contradiction might be explained by different experimental and biological conditions: i) the use of a constitutive membrane-bound AKT mutant [38], [37] compared to the ^E545K^PIK3CA and the ^delE746*-*A750^EGFR oncogenes; ii) the endogenous expression levels of the oncogenic allele in the knock-in models compared with the constitutive transgenic over-expression; iii) tissue-specific metabolic effects of each cancer mutations; iv) the use of pre-cancerous cells instead of transformed cancer cells that could, indeed, carry on additional mutations and whose combination could generate more complex metabolic phenotypes.

The observed oncogene-induced resistance to glucose deprivation in epithelial cells indicates that, during neoplastic progression, cancer cells may display a variable degree of glucose addiction, depending on the tumor stage: advanced tumors may show an increased glycolytic rate and glucose addiction as adaptive strategies that support acidosis, hypoxic growth and invasion. We suggest that pre-cancerous cells have an opposite strategy, since glucose addiction represents a strong metabolic Achilles' heel that limits the progression of cells carrying a functional AMPK, which represents an important metabolic checkpoint controlling cell fate under glucose deprivation [14]. In this perspective, glucose availability represents an intrinsic barrier that restricts aberrant proliferation of mammary cells. Oncogenic activation of EGFR or PI3K pathways selects and drives cellular clones able to surmount such metabolic barriers and to survive under sub-optimal microenvironment conditions.

### Enhanced nuclear β-catenin and FOXO4 signalling by oncogenic EGFR and PIK3CA

We have documented a positive feedback between the oncogenic EGFR and PI3K pathways with the FOXO4 and β-catenin signals in response to glucose deprivation. The role of FOXO proteins as tumor suppressors has been largely recognized and has been associated with their ability to promote cell cycle arrest [42]. Here, we propose that FOXO4, specifically activated by β-catenin, promotes cell resistance to oxidative metabolic stress and survival of oncogenes-carrying cells. This dual function of FOXOs may depend on a complex code of post-translational modifications and interacting co-activators that differentially control FOXOs functions under different conditions and in a tissue specific manner [42], [43]. There is evidence indicating an evolutionary conserved interaction between FOXO4 and β-catenin induced by starvation and enhanced by oxidative stress that drives the expression of antioxidant enzymes, such as MnSOD [26]. Along with these observations, our findings support the conclusion that β-catenin is a transcriptional co-activator that switches on FOXO target genes under nutrient stress and promotes cell survival.

We show that somatic mutations frequently observed in breast cancer lead to β-catenin activation: this observation strengthens emerging data outlining the relevance of the β-catenin activation in breast cancer [44], [45] and the crosstalk between EGFR and WNT signals in breast cancer development [46]. One mechanism, at least, involves the regulation of GSK3β, a key kinase controlling β-catenin stability. We propose that the GSK3β, β-catenin and MnSOD axis represents a potential target to lower the resistance to oxidative stress of tumor harboring oncogenic EGFR and PI3KCA.

## Materials and Methods

### Plasmids

Flag-β-catenin, pMT2-HA-FOXO4, pSOD-luc(−3340+1) and the pSODmut-luc carrying point mutations in two FOXO binding sites were a generous gift from Dr B.M. Burgering (University Medical center, Utrecht, The Netherlands) and have been described elsewhere [24], [26]. CMV-Renilla luciferase was purchased from Promega. The pCDNA-MnSOD has been previously described [47]. Expression plasmids for wild type GSK3β and dominant-negative GSK3β(K85A) were purchased from Addgene.

### Cell viability

Cell viability was determined by flow cytometry using propidium iodide (PI) staining. Briefly, after the specific treatments, detached and attached cells were collected, washed twice with PBS 1× and stained with propidium iodide; PI positive cells, e.g. death cells, were detected by flow cytometry with a FACSCalibur (Becton Dickinson) and analyzed by using the CellQuest software (Becton Dickinson).

### ATP quantification

Cells were counted and plated in a 96-well plate in quadruplicates at the density of 3000 cells/well. After 48 hours, cells were glucose-starved and harvested at different time points. ATP assay was carried out using the ATPlite assay (Perkin Elmer) according to the manufacturer's instructions. A parallel experiment was performed to determine the cell number by nuclei counting after staining with 1 µg/ml Hoechst 33342. Nuclei were detected by florescence microscopy with a BD pathway HT bioimager with the AttoVision Acquisition Software Module and quantified by using the BD Date Image Explorer Software.

### GSH/GSSG measurement

Reduced and oxidized Glutathione ratio was measured by using the GSH/GSSG-Glo assay kit (Promega) according to the manufacturer's protocol.

### Quantitative Real-Time PCR

Total RNA extraction was done using Tryzol (Invitrogen) according to the manufacturer's instructions. Total RNA was then reverse-transcribed into cDNA by using M-MLV Reverse Transcriptase (Gibco BRL) with oligo random hexamers. The cDNA was subjected to quantitative PCR analysis by using Light Cycler (Applied Biosystem) with SYBR Green PCR Master MIX Kit (Applied Biosystem). The primers sequences for the PCR analysis are available on request.

### Statistical analysis

Data are presented as mean ± standard deviation (SD). Statistical significance was analyzed by using, where appropriate, a two-tailed Student's *t*-test. P values less or equal than 0.05 were considered statistically significant.

Additional experimental procedures (materials and reagents, cell lines, cell culture and transfection, protein extracts, western blot analysis, glycogen measurement, luciferase reporter assay and immunoflorescence analysis) are provided as Methods S1.

## Supporting Information

Figure S1 **The insertion of oncogenic alleles by homologous recombination (knock-in) effectively and specifically affects the downstream signaling pathways in mammary epithelial cells.** Wild type HME or isogenic cells carrying *^delE746-A750^EGFR* or *^E545K^PI3KCA or ^G13D^KRAS* cancer mutations were treated with the indicated serum concentrations for 16 hours. Equal amounts of total protein extracts were analyzed by immunoblot with the indicated antibodies. ERK1/2 indicates the p42/p44 proteins; pERK1/2 indicates the Thr202 and Tyr204 phosphorylated residues of ERK1/2. pMEK1/2 indicates the phosphorylated residues at Ser217 and Ser221 of MEK1/2. Data are representative of three independent experiments that gave similar results.
(TIF)

Figure S2 **HME clones carrying the** ***^delE746-A750^EGFR*** **or the** ***^E545K^PIK3CA*** **allele are resistant to GD-induced cell death.** Additional HME clones carrying oncogenic mutations - independently generated from clones presented in Figure 1 - were glucose starved for 48 hours. The percentage of dead cells was quantified by FACS analysis of propidium iodide positive cells. Results report the data derived on the average from four independent experiments ± SD.
(TIF)

Figure S3 **Phosphorylation of AMPKα(T172) in wild type HME cells and in isogenic control knock-in cells.** (A) Additional HME clones carrying oncogenic mutations - independently generated from clones presented in Figure 1 - were glucose starved for 10 hours and equal amount of total protein extracts were assayed by immunoblot with the indicated antibodies. (B) Wild type HME and isogenic knock-in clones generated by homologous recombination of the wild type *EGFR* or *PIK3CA* alleles were treated and analyzed as in (A). The levels of pAKT(S437) on the same protein extracts are also reported showing that the activation of the PI3K-dependent pathways is comparable in all three clones.
(TIF)

Figure S4 **Upregulation of MnSOD by** ***EGFR*** **or** ***PIK3CA*** **cancer alleles in response to GD.** Wild type HME and isogenic cells carrying *^delE746-A750^EGFR* or *^E545K^PIK3CA* alleles were glucose starved for the indicated hours. Total proteins were extracted and analyzed by immunoblot with the indicated antibodies. The graph reports the densitometry analysis of the MnSOD/TOM1 signals and the average from three independent experiments ± SD.
(TIF)

Methods S1 (DOC)
