# Supplementary figures and images for: Activation of β-Catenin by Oncogenic PIK3CA and EGFR Promotes Resistance to Glucose Deprivation by Inducing a Strong Antioxidant Response
Source: PLoS One. 2012 May 25;7(5):e37526. doi: 10.1371/journal.pone.0037526 (PMC3360841; doi:10.1371/journal.pone.0037526)

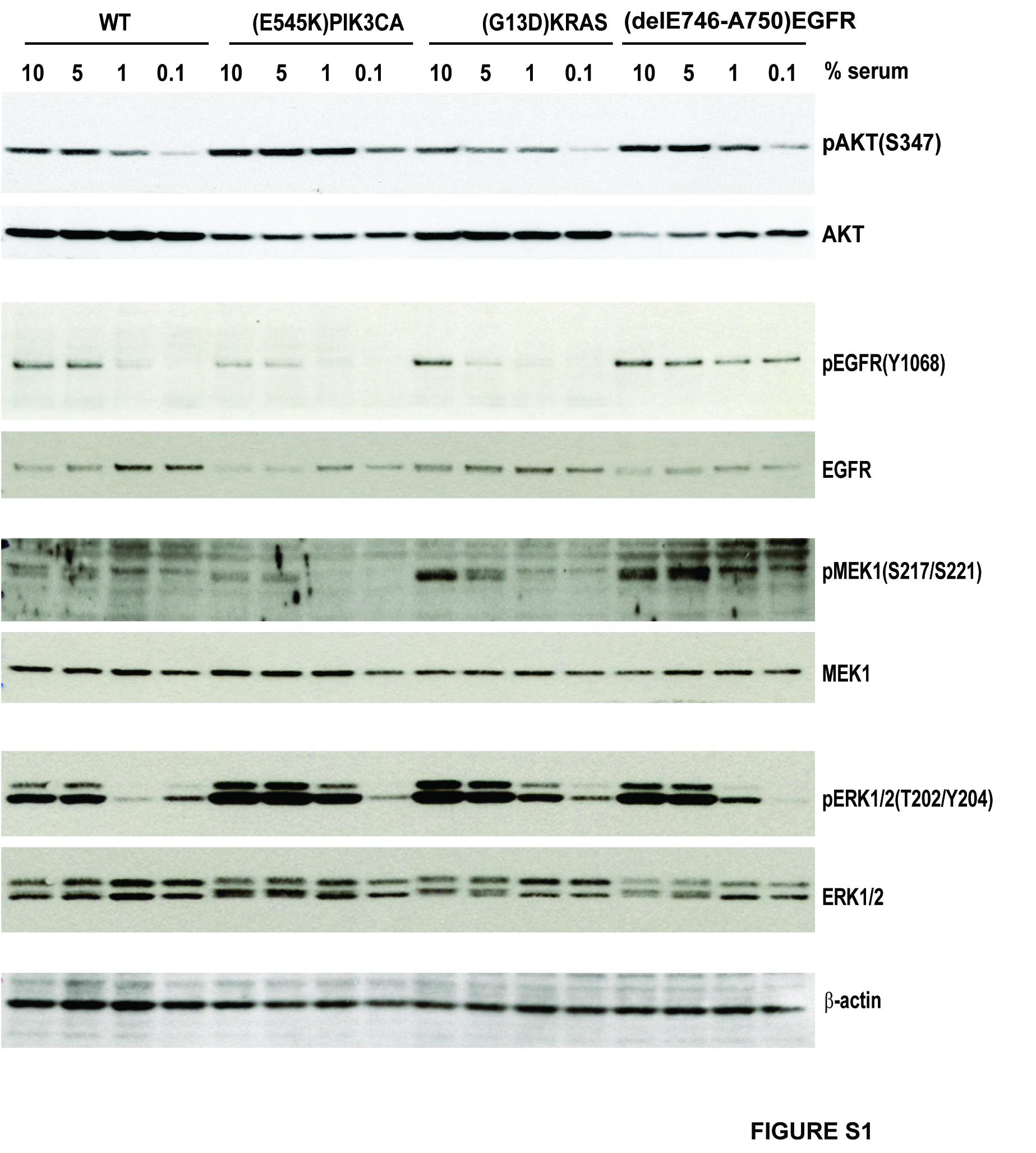

Supplement: Figure S1 — The insertion of oncogenic alleles by homologous recombination (knock-in) effectively and specifically affects the downstream signaling pathways in mammary epithelial cells. Wild type HME or isogenic cells carrying delE746-A750EGFR or E545KPI3KCA or G13DKRAS cancer mutations were treated with the indicated serum concentrations for 16 hours. Equal amounts of total protein extracts were analyzed by immunoblot with the indicated antibodies. ERK1/2 indicates the p42/p44 proteins; pERK1/2 indicates the Thr202 and Tyr204 phosphorylated residues of ERK1/2. pMEK1/2 indicates the phosphorylated residues at Ser217 and Ser221 of MEK1/2. Data are representative of three independent experiments that gave similar results. (TIF) [file pone.0037526.s001.tif]

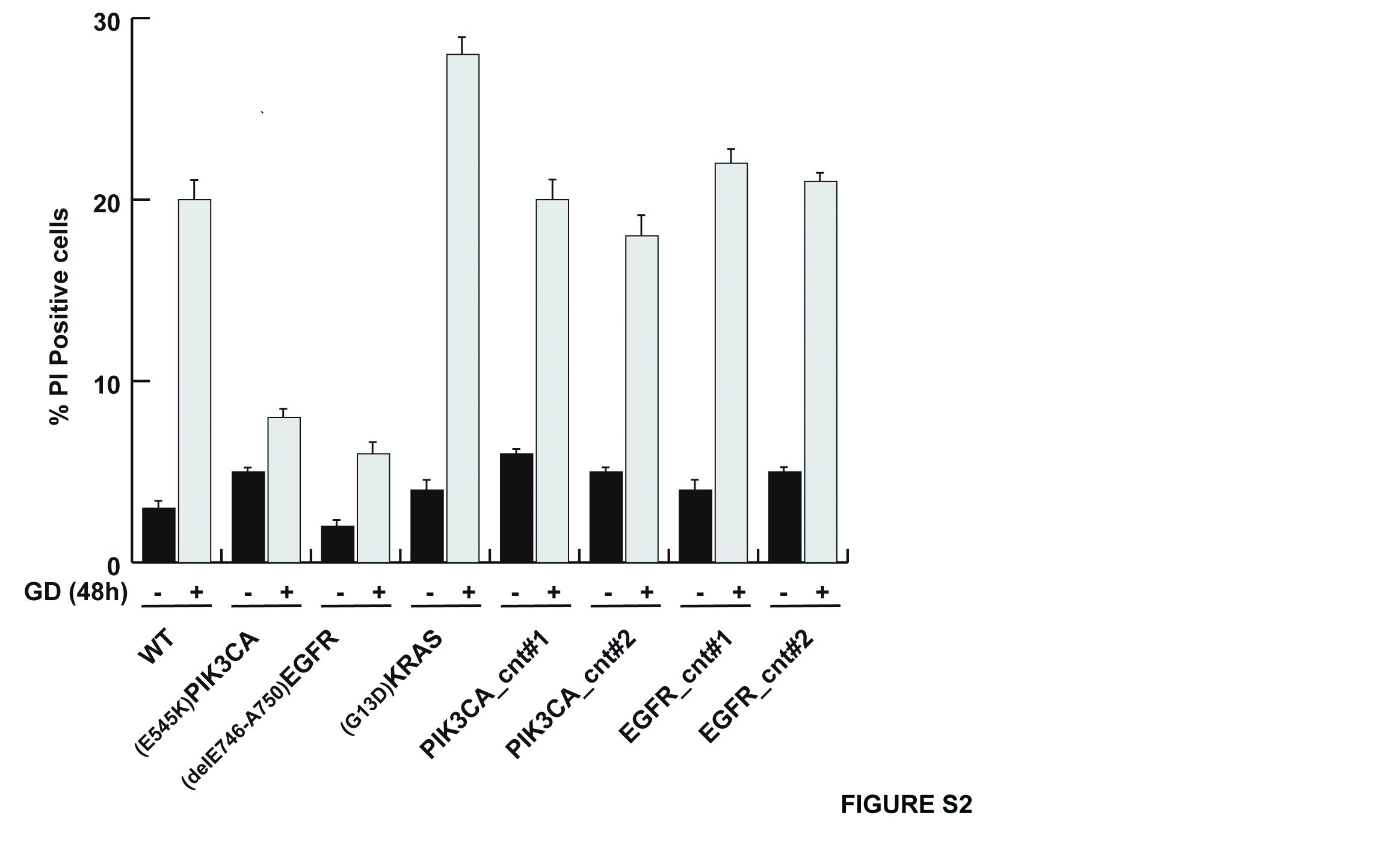

Supplement: Figure S2 — HME clones carrying the delE746-A750EGFR or the E545KPIK3CA allele are resistant to GD-induced cell death. Additional HME clones carrying oncogenic mutations - independently generated from clones presented in Figure 1 - were glucose starved for 48 hours. The percentage of dead cells was quantified by FACS analysis of propidium iodide positive cells. Results report the data derived on the average from four independent experiments ± SD. (TIF) [file pone.0037526.s002.tif]

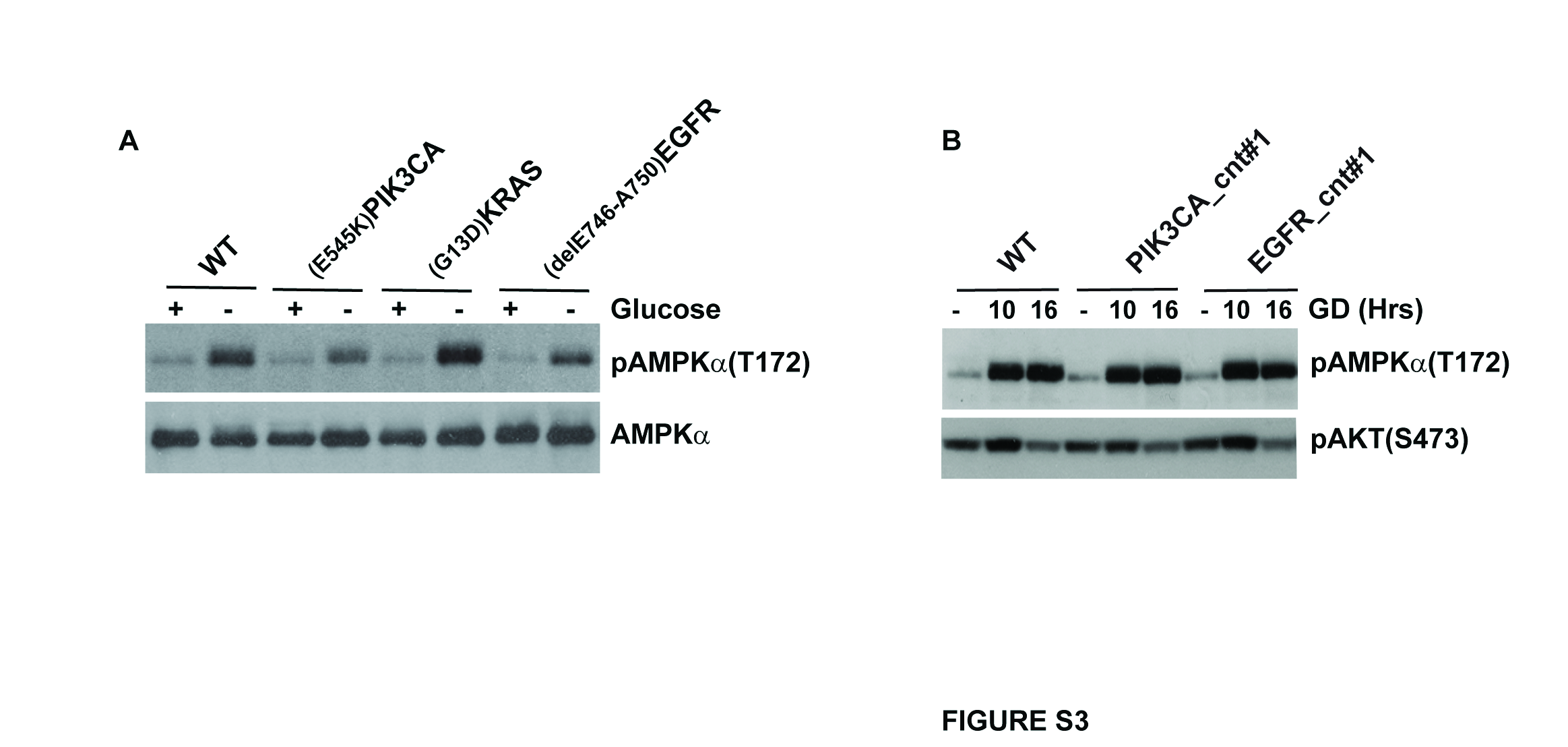

Supplement: Figure S3 — Phosphorylation of AMPKα(T172) in wild type HME cells and in isogenic control knock-in cells. (A) Additional HME clones carrying oncogenic mutations - independently generated from clones presented in Figure 1 - were glucose starved for 10 hours and equal amount of total protein extracts were assayed by immunoblot with the indicated antibodies. (B) Wild type HME and isogenic knock-in clones generated by homologous recombination of the wild type EGFR or PIK3CA alleles were treated and analyzed as in (A). The levels of pAKT(S437) on the same protein extracts are also reported showing that the activation of the PI3K-dependent pathways is comparable in all three clones. (TIF) [file pone.0037526.s003.tif]

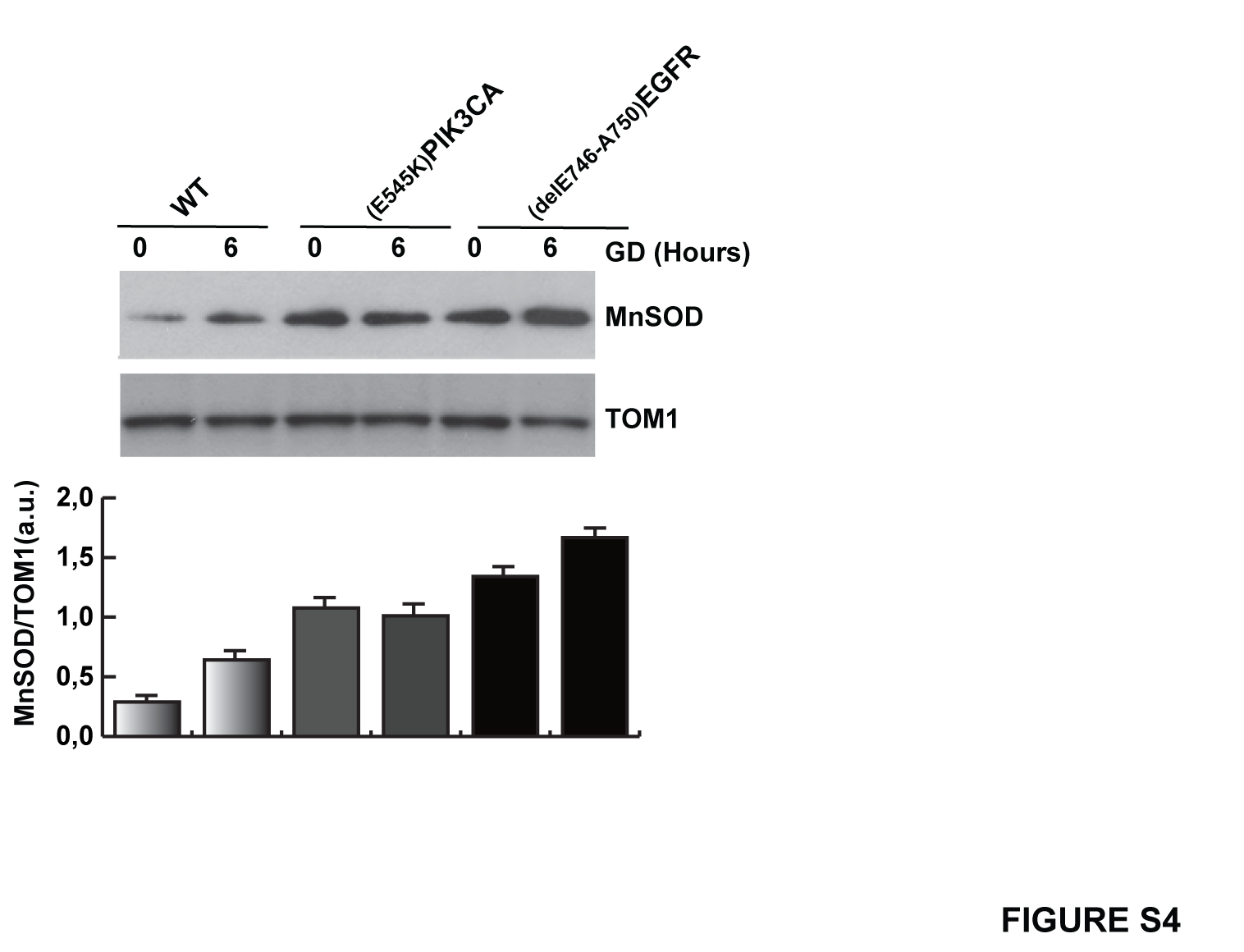

Supplement: Figure S4 — Upregulation of MnSOD by EGFR or PIK3CA cancer alleles in response to GD. Wild type HME and isogenic cells carrying delE746-A750EGFR or E545KPIK3CA alleles were glucose starved for the indicated hours. Total proteins were extracted and analyzed by immunoblot with the indicated antibodies. The graph reports the densitometry analysis of the MnSOD/TOM1 signals and the average from three independent experiments ± SD. (TIF) [file pone.0037526.s004.tif]
