# Supplementary material for: Activation of β-Catenin by Oncogenic PIK3CA and EGFR Promotes Resistance to Glucose Deprivation by Inducing a Strong Antioxidant Response
Source: PLoS One. 2012 May 25;7(5):e37526. doi: 10.1371/journal.pone.0037526 (PMC3360841; doi:10.1371/journal.pone.0037526)
Supplement: Methods S1 — (DOC) [file pone.0037526.s005.doc]

**Methods S1**

**Materials and Reagents**

The following antibodies were purchased as indicated and used according manufacture’s instructions: anti-phospho-AMPKα(Thr172), anti-AMPKα, anti-phospho-ERK1/2(Thr202/Tyr204), anti-ERK, anti ERK1/2, anti-phospho-MEK1/2, anti-MEK1/2, anti-phosphoAKT(Ser473), antiAKT, anti-phosphoLKB1(Ser428), anti-LKB1, anti-phosphoACC1(Ser79), anti-ACC1, anti-phosphoEGFR(Tyr1068), anti-EGFR, anti-phospho-β-catenin(ser33,37,Thr41), anti-GSK3β, the anti-phosphoGSK3β(Ser9) and the anti-FOXO1 antibodies were purchased from Cell Signaling Technology. Anti-β-catenin and anti MCM7 antibodies were from Santa Cruz. Anti active, not phosphorylate Ser37/Thr41 β-catenin and anti MnSOD antibodies was from Millipore. The anti-β-actin antibody was from Sigma-Aldrich. The anti-HA antibody was from Covance inc. The anti-FOXO4 antibody was a generous gift from Dr. Burgering’s lab (University Medical center, Utrecht, The Netherlands). D-Glucose, N-Acetyl-L-Cysteine, human recombinant catalase, AICAR, Lithium Chloride, 6AN, and CP91149 were purchased from Sigma-Aldrich. Sodium pyruvate was from Gibco. The cell permeable SOD mimetic (MnTMPyP) was purchased from Calbiochem.

**Cell lines, cell culture and transfection**

Mammary epithelial hTERT-HME1 (HME) cell lines and isogenic derivatives carrying specific cancer alleles generated by adeno-associated virus (AAV)-mediated targeted homologous recombination have been described previously [1,2]. HME cells were cultured in growth medium containing RPMI (Invitrogen) supplemented with 20 ng/mL EGF (Sigma-Aldrich), 10 μg/ml insulin (Sigma-Aldrich), and 100 μg/ml hydrocortisone (Sigma-Aldrich), 5% FBS (Invitrogen), 1mM Glutamine (Invitrogen), 50 units/ml penicillin and 100 μg/ml streptomycin (Invitrogen). Glucose starvation was carried out by starvation media: Glucose-free RPMI supplemented with 20 ng/mL EGF, 10 μg/ml insulin, and 100 μg/ml hydrocortisone, 5% dialyzed FBS serum (10000 Dalton C.O, Invitrogen), 50 units/ml penicillin and 100 μg/ml streptomycin. All hormones were diluted in Glucose free-RPMI media. The glucose content, measured by standard kit, was confirmed as undetectable by using Glucose free-RPMI media as reference. Control cells (e.g. +Glucose) were treated identically except that growth media was supplemented with 4.5 g/L D-Glucose. Serum starvation was carried out by using serial dilutions in RPMI supplemented with glutamine and antibiotics. For starvation experiments, cells were counted, plated and grown for 3 days in complete media. Then cells were washed twice with starvation media before starting starvation. All experiments with cell lines were performed between passages 2 and 4.

HME cells were transiently transfected using the TransIT-LT1 reagent (Mirus) according to the manufacturer. Total amount of DNA were equalized using pBluscript-KS vector. For stable transfection, cells were transfected using the TransIT-LT1 reagent (Mirus) and growh under G418 antibiotic. Two week after, single clones were isolated, expanded and analyzed by RT-PCR and immunoblot analysis for target gene expression.

**Protein extracts and western blot analysis**

Cells were washed in phosphate-buffered saline 1X (PBS1X) containing 1X protease inhibitor cocktail and 1mM PMSF and were lysated in modified radioimmunoprecipitation assay buffer (RIPA-1: 50 mM Tris-HCl [pH 7.8], 150 mM NaCl, 5 mM EDTA, 15 mM MgCl2, 1% Nonidet P-40,

0.5% sodium deoxycholate, 1 mM dithiothreitol, 1X protease inhibitors, 1mM PMSF, 50mM NaF, 10 mM β-glycero-phosphate and 1 mM Na_3_VO_4_, and then immediately frozen in liquid nitrogen.

Nuclear proteins extracts were prepared as previously described [3]. Cleared proteins extracts were quantified by using the Bradford method (Bio-Rad). For western blotting, protein samples were separated on 8%–12% SDS-PAGE and transferred to nitrocellulose membrane (Amersham). Membranes were blocked in TBS containing 5% bovine serum albumin, incubated with primary antibodies according to the antibody manufacturer's instructions, followed by incubation with horseradish peroxidase-conjugated goat anti-rabbit or anti-mouse IgG (Amersham) and enhanced chemiluminescence detection (Pierce). Band intensity (area x density) of the immunoblot was analyzed and quantified using the NIH1.6 image software. Background was subtracted from the quantification area.

**Total Glycogen measurement**

Total cellular glycogen was isolated and quantified according with the amylo-α-1,4-α-1,6-glucosidase digestion method [4]. Glycogen amount was quantified as released free glucose with the glucose assay kit (Sigma-Aldrich) according to the manufacturer’s instructions.

**Luciferase reporter assay**

HME cells were cotransfected with HA-FOXO4, HA-FOXO3a, or control plasmid. After 20 hour, cells were washed and glucose starved for 8 hours. For the luciferase assay, cells were washed twice with phosphate-buffered saline, lysed in passive lysis buffer and luciferase activity was analyzed using a luminometer and a dual-luciferase assay kit according to the manufacturer (Promega). The fold induction of luciferase activity on the control cells (e.g. no luciferase vector) was plotted. The efficiency of transfection was normalized by the cotransfection of CMV-Renilla luciferase reporter.

**Immunoflorescence analysis**

Cells were cultured on chamber Polystyrene vessel tissue culture treated glass slides (BD Falcon). 48 hours after plating cells were treated or not as indicated and were rinsed in phosphate-buffered saline (PBS) and fixed with 3.7% formaldehyde/PBS for 30 min at room temperature. Cells were treated with Blocking Agent (BA) (0.05% saponin/0.5% BSA/PBS) for 30’ and incubate 1 hour in BA with primary anti active β-catenin antibody. After 3 washes with PBS, cells were incubated for 30 min with goat anti-rabbit IgG conjugated to the fluorescent Alexa 495 dye (1:1000, Invitrogen Molecular Probe). After 3 washes, samples were DAPI stained and mounted in Vectashield Medium (Vector Laboratories Inc, Abcys, Paris, France) for viewing with a fluorescence microscope (FV 1000 Olympus IX-81).

Additional references

1. Arena S, Isella C, Martini M, de Marco A, Medico E, et al. (2007) Knock-in of oncogenic Kras does not transform mouse somatic cells but triggers a transcriptional response that classifies human cancers. Cancer Res 67: 8468-8476.

2. Di Nicolantonio F, Arena S, Gallicchio M, Zecchin D, Martini M, et al. (2008) Replacement of normal with mutant alleles in the genome of normal human cells unveils mutation-specific drug responses. Proc Natl Acad Sci U S A 105: 20864-20869.

3. Hoppe-Seyler, F., Butz, K., Rittmuller, C., and von Knebel Doeberitz, M. (1991). A rapid microscale procedure for the simultaneous preparation of cytoplasmic RNA, nuclear DNA binding proteins and enzymatically active luciferase extracts. Nucleic Acids Res 19, 5080.

4. Lust WD, Passonneau JV, Crites SK (1975) The measurement of glycogen in tissues by amylo-alpha-1,4-alpha-1,6-glucosidase after the destruction of preexisting glucose. Anal Biochem 68: 328-331.
